# Evaluation of the Ethical Controversies in Separation Surgery of Conjoined Twins: A Review of the Literature

**DOI:** 10.7759/cureus.77382

**Published:** 2025-01-13

**Authors:** Yan Ting Woo

**Affiliations:** 1 Oncology, Leeds Teaching Hospitals NHS Trust, Leeds, GBR

**Keywords:** conjoined twins, decision making process, ethical dilemma, separation surgery, siamese twins, surgical ethics

## Abstract

This study aimed to evaluate the ethical controversies surrounding separation surgery of conjoined twins by examining the discussions in the relevant published literature. A critical review was conducted to study the ethical issues involved in the separation surgery of conjoined twins. A literature search on PubMed and Embase was performed on December 4, 2023, using the terms "ethics", "conjoined twins", and "separation". Relevant papers published in the last 10 years (2013-2023) were included. A total of seven papers were included: two were case reports and five were expert statements or opinions. The four pillars of ethics (beneficence, non-maleficence, autonomy, and justice) were a common approach adopted by most authors to analyse the ethical issues in the separation surgery of conjoined twins. Due to the need to consider the perspectives of two patients simultaneously, opposing arguments were common depending on the approach taken, especially when analysing the principles of beneficence and non-maleficence. All papers examined the possible difficulties in autonomy, with some exploring consent, specifically around decision-making. It was largely agreed that the parents should be the decision-makers due to the patients’ lack of capacity, except in non-urgent situations, where patients should be given the opportunity to make their own decisions. Achieving equality in the distribution of shared organs and survival opportunities proved challenging. Other ethical principles were also discussed, such as the act-omission and double effect doctrines. Limitations of this paper included the limited geographical coverage of the studies, as well as the potential to miss relevant studies, including those conducted beyond the search period, those published in other languages, and those unpublished. From the overall results, there is no apparent definite answer as to whether separation surgery is justified. Due to every patient and situation being unique, the best approach should be decided on a case-by-case basis, guided by the four pillars of ethics, and with the involvement of families and ethical committees. Both the ethical considerations and other factors should be taken into account.

## Introduction and background

Conjoined twins are rare anomalies that have been a significant topic of interest due to their rarity, the challenging management, and the ethical dilemmas involved. They can be categorised into symmetrical and asymmetrical twins [1], with the latter further classified based on the site of fusion, such as thoracopagus (conjoined at the chest), craniopagus (attached at the head), and omphalopagus (joined at the abdomen) [2-4]. With technological advancements in medicine, prenatal diagnosis has become possible [3,4]. For mothers who choose to continue with the pregnancies and where the twins survive delivery, there are three main management options: no surgical intervention, immediate surgery post-delivery (i.e., emergency separation), or surgery at a later stage [3,4]. Post-surgical survival rates vary depending on the type of fusion involved [2,3].

The challenges in the separation surgery of conjoined twins lie not only in the practical aspects of the surgery itself but also in the ethical concerns raised in such cases. Many existing published studies discuss the possible causes, diagnosis, and management of conjoined twins [2,5]. However, there is a lack of focus in these articles on the ethical issues, as well as a shortage of studies addressing the ethical dilemmas involved. This study aims to examine and summarise the ethical concerns in the separation surgery of conjoined twins by analysing the relevant discussions in the published literature. It is hoped that this analysis will provide a foundation for healthcare professionals dealing with similar cases, enabling more comprehensive planning and better anticipation of possible ethical difficulties.

This article was previously presented as an oral e-poster at The Association of Surgeons in Training 48th Annual Conference on March 9, 2024.

## Review

Method

**Figure 1:**
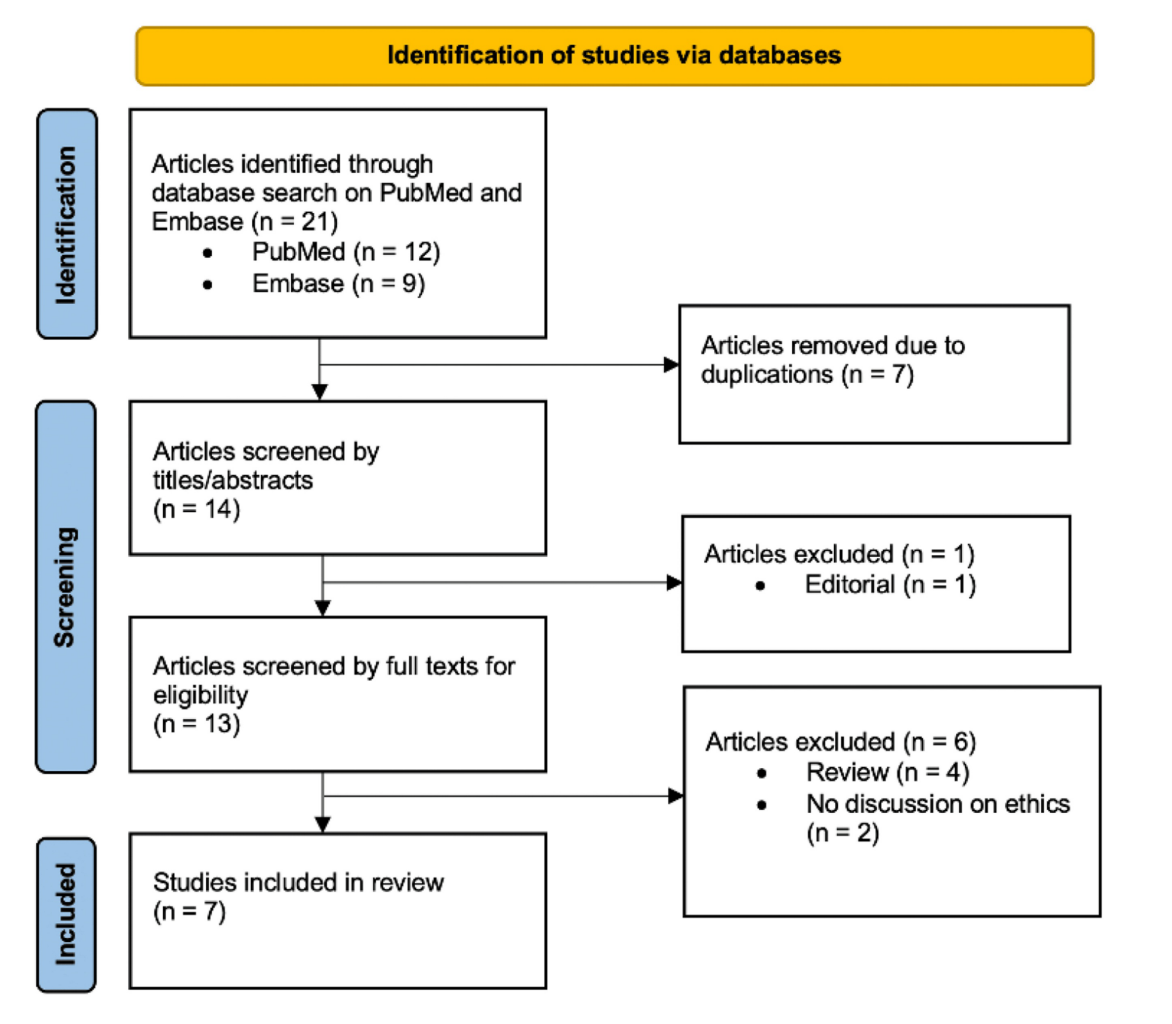
Flowchart of study selection process. Adapted from the Preferred Reporting Items for Systematic Reviews and Meta-Analyses (PRISMA) 2020 Flow Diagram [5].

A critical review of the ethical issues surrounding the separation surgery of conjoined twins was conducted on December 4, 2023, following the Preferred Reporting Items for Systematic Reviews and Meta-Analyses (PRISMA) standard [6]. The electronic databases PubMed and Embase were searched for relevant articles using three search terms: "ethics", "conjoined twins", and "separation". The publication timescale was limited to the past 10 years (2013-2023) to ensure the retrieval of the most recent and relevant publications. All results were in English. Duplications, review papers, editorial papers, and papers that did not include discussions on ethics were excluded. This process resulted in seven papers, all of which were included and analysed in this review to identify the key concepts discussed. These concepts were then categorised into major themes. The selection process of the relevant studies is summarised in Figure 1.

Results

**Figure 2:**
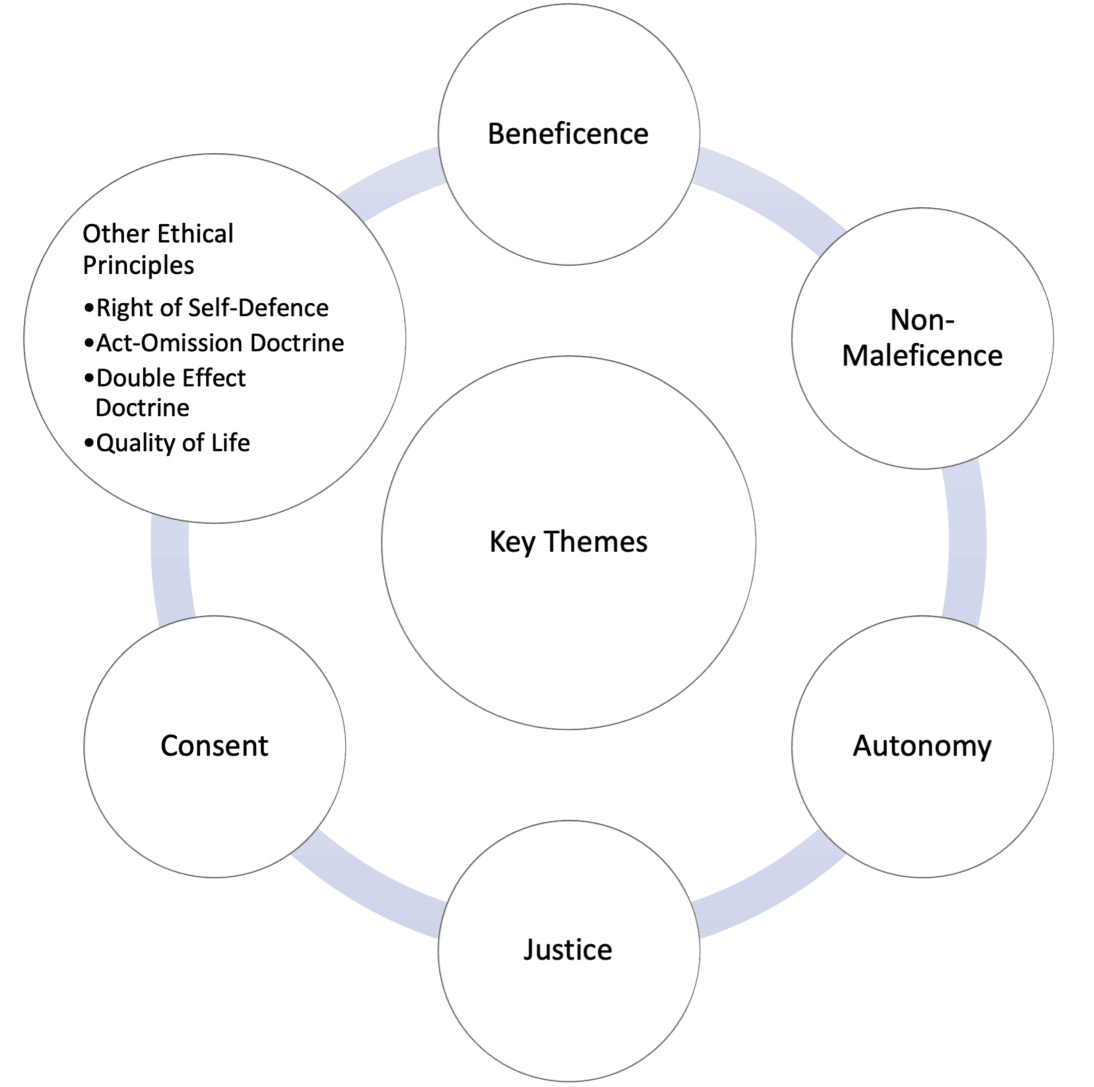
Summary of the key ethical themes discussed in the included papers.

The search yielded 12 papers on PubMed and nine on Embase. After applying the above-mentioned exclusion criteria, seven papers were included in this review, as shown in Table 1 [7-13]. These comprised two case reports and five expert statements or opinions. Most papers addressed the ethical issues encountered in the separation surgery of conjoined twins using the framework of the four pillars of ethics, with some also exploring other ethical principles (Figure 2).

**Table 1:** Overview of all seven papers included in this review.

Paper	Year Published	Country Published	Ethical Themes Discussed					
			Beneficence	Non-maleficence	Autonomy	Justice	Consent	Other Ethical Principles
Ramlan et al. [7]	2023	Indonesia	Yes	Yes	Yes	Yes	Yes	No
Harvey et al. [8]	2018	United States	No	Yes	Yes	No	Yes	No
Cummings et al. [9]	2018	United States	Yes	Yes	Yes	No	No	Yes: double effect doctrine
Kallberg [10]	2018	United States	No	Yes	Yes	No	Yes	Yes: quality of life
Savulescu et al. [11]	2016	United Kingdom	No	Yes	Yes	Yes	Yes	Yes: right of self-defence act-omission doctrine double effect doctrine
Spitz [12]	2015	United Kingdom	No	No	Yes	Yes	Yes	No
Davis [13]	2014	Australia	No	No	Yes	Yes	Yes	Yes: act-omission doctrine

Themes One and Two: Beneficence and Non-maleficence

The themes of beneficence and non-maleficence are commonly intertwined and analysed together, as they involve achieving the right balance between acting in the patient’s best interest and avoiding harm. In the case of conjoined twins, there are two patients to consider simultaneously. Where separation surgery benefits Twin A, there is a possibility of causing harm to Twin B, and vice versa [7-9]. The dilemma may be less pronounced when emergency separation is warranted or when separation is unavoidable to ensure the survival of at least one twin, with the potential deaths of both twins as a possible outcome if no action is taken. However, this does not negate the recognition of harm caused to the other twin [7]. Kallberg also argued that if separation, despite being an emergency due to the deterioration of one twin, is performed while both twins are alive, it could be equivalent to the act of killing [10].

Beyond survival, the ethics of non-maleficence must also be considered from other perspectives, including quality of life and various social factors. Where significant organ overlaps or anomalies are present, Savulescu et al. opposed separation, arguing that it might threaten the survival of one or both twins or result in a suboptimal quality of life, thereby causing harm. However, remaining conjoined might lead to social discrimination or the fatality of one twin when the other’s life is under threat. They suggested that whenever separation surgery is considered, the potential benefits should always be weighed against the likely post-surgical prognosis and quality of life [11].

Themes Three and Five: Autonomy and Consent

All seven papers explored the ethical topic of autonomy. This is specifically with regard to the decision-maker(s), mainly the patients (i.e., the twins), the parents, and the court. As the ethics of consent is closely related to autonomy, both topics will be discussed together in this section.

In cases where the twins lack capacity and moral dilemmas are present, the parents are usually given the autonomy to make the decisions [7,9,12]. They should be involved in all stages of the discussions and care, with sufficient information provided to allow fully informed decision-making [9]. With good communication and consistent involvement of the parents, reaching a consensus between them and the medical team is possible, as evident in Harvey et al.’s reported case [8]. On the other hand, Ramlan et al. raised an example where insufficient information was given during the pregnancy regarding the twins’ probable viable survival and separation surgery, which might have impacted the subsequent decision-making processes, thus reiterating the importance of providing information and obtaining informed consent, regardless of the likelihood of potential events [7].

The opinions of the extended families and religious leaders should also be considered, especially in cultures where strong emphasis is placed on religion and other social factors. Ramlan et al. suggested that these individuals’ opinions should be respected despite them being non-legally binding [7]. While respected figures’ opinions should be considered, issues might arise when a consensus cannot be reached. In Ramlan et al.’s reported case of female thoraco-omphalopagus twins, the medical team suggested medical management of Twin B’s neonatal sepsis, but the parents and a respected family figure insisted on surgery, which was eventually performed. Unfortunately, both twins passed away due to different post-operative reasons [7].

In situations where agreement cannot be reached or where further support is needed, the court might be involved. In Farah and Saba’s case, the court claimed that it has the duty to protect one twin’s right to life despite the possibility of going against parental wishes [13]. It is worth noting that in this case, separation surgery did not proceed due to the lack of medical evidence to warrant the surgery, raising questions about the appropriateness of court involvement. From an objective perspective, Spitz et al. argued that courts might be better positioned to make the best decision for the dignity of the patients by applying "principled reasoning" [12].

With regard to patients’ autonomy and consent, there are additional points to consider. For older patients who might be able to consent to medical treatments, the law and practice vary between countries. For example, in the United Kingdom and Australia, children under the ages of 16 and 18, respectively, might be able to consent to medical treatments if deemed competent according to Gillick competency [14,15]. However, in India, the application of Gillick competency is less clear, with the general age of consent being 18 [13,16]. As a result, parents or families might take on a greater role in decision-making in these situations. This was evident in Farah and Saba Shakeel’s case, where their preferences were not mentioned throughout the court proceedings [13].

Furthermore, Savulescu et al. and Spitz argued that current evidence has shown that many twins prefer to remain conjoined, and decision-making should therefore be deferred until they have capacity, wherever possible [11,12]. This will, however, be at the expense of their time to adapt to their self-image [12]. "Substituted judgment" and "hypothetical consent" could be used as guidance to consider the likely preference of the twins and their decisions if they were competent, but these should not be used as justifications [9,11].

The debate becomes more challenging when interpreting autonomy in terms of the twins’ rights to their lives and shared organs, especially in parasitic twins. This is seen in the case of Mary and Jodie, where authors held opposing views when considering the issue from each of the twins’ viewpoints. Three papers argued that the healthier twin (Jodie) had the right of self-defence and the right to refuse the altruistic act of supporting the parasitic twin (Mary), which would shorten her survival [9,11,12]. On the other hand, Kallberg argued that with the blood being a shared entity, both twins should have equal rights to it [10]. Furthermore, they argued that one should balance the right to self-defence against the parasitic twin’s (i.e., the "attacker’s") right to life and consider if overriding factors exist for either stance [10].

It is worth noting that while most would argue for sacrificial separation to reduce the risk of death for one twin, Kallberg opposed this reasoning based on the argument of involuntary lethal amputation or transplantation [10]. They argued that, in conjoined twins, organs are shared without distinct boundaries, and separation surgery could therefore be considered involuntary amputation surgery (due to the patient’s lack of capacity to consent) with death as a possible severe risk, or even involuntary transplant surgery involving the reassignment of organs [10].

Theme Four: Justice

Four papers discussed the ethical pillar of justice, mainly focusing on equality in the distribution of shared organs and survival opportunities. Where separation is needed, efforts should be made to ensure the equal distribution of organs and survival chances as far as possible [12], which may not be straightforward, especially when there is unequal allocation between the twins to begin with [7]. Savulescu et al. analysed this issue based on the argument of natural inequality, where the twins are healthier and disadvantaged by chance, respectively, and this natural inequality could be exacerbated through separation surgery [11]. However, they noted that this argument, as well as the ownership rights to the organs, could potentially be overridden by the extent of the respective potential benefits and harms, including the prognosis [11]. These gains and losses should also be balanced while considering the least harmful option in sacrificial separation surgery [13]. It is worth noting that such weighting and balancing might be instinct-based, given the absence of definitive right or wrong answers [11].

Other Ethical Principles

Besides the aforementioned pillars of ethics, other ethical principles were also raised by the authors.

Right of self-defence: The right of self-defence has been used as an argument for separation, as discussed above. This is especially relevant in parasitic twins, where one twin acts as the "unjust aggressor" by relying on the other twin for survival [9]. However, the role of third parties in using self-defence as an argument is debatable, as self-defence should be initiated by the victims themselves, rather than by others [11]. The dilemma around organ distribution would also persist despite the acceptance of this self-defence argument [11].

Act-omission doctrine: The controversies surrounding acts of omission and active killing have long existed in ethical debates, such as those on euthanasia, and the discussion in conjoined twins is no exception. Davis argued that the positive act of performing separation surgery, which results in the death of one twin, might be permitted by the courts if there is no other alternative to save the life of at least one of them [13]. However, some believe that, compared to the act of killing, the act of omission (by not performing the separation surgery) might be less challenging to justify morally, even though it could lead to the eventual death of both twins [11].

Double effect doctrine: The double effect doctrine should always be considered alongside the act-omission doctrine. The harm caused during separation surgery might be justifiable if it is a foreseeable side effect rather than the intended means or end result of the surgery [9,11].

Quality of life: The concept of quality of life has been addressed in various discussions above, particularly under the topics of autonomy and decision-making. Most considerations of quality of life focus on the post-separation period to determine if the surgery is justifiable. However, quality of life should also be analysed when the twins are conjoined, using the internalist and externalist theories [10]. Some may argue for separation surgery to benefit the autonomy and freedom of both twins. However, Kallberg argued that these constraints may not necessarily be negative, as the fusion could also bring internal benefits to the twins, such as irreplaceable bonds and intimacy [10]. It should be noted that (a) this might not apply universally to all conjoined twins and should therefore be assessed on a case-by-case basis, and (b) it may not be applicable in cases where sacrificial separation is deemed necessary due to the high risk of fatality involved [10].

Discussion

This literature review provides an overview of the current ethical dilemmas surrounding the separation surgery of conjoined twins, which have been analysed based on the respective major themes. This could help form the foundation for discussions when healthcare professionals are considering separation surgery for their patients and also for future research.

There are a few limitations to this review. Firstly, the search was limited to the years from 2013 to 2023 to identify the more recent ethical dilemmas. As a result, earlier ethical concerns, which might still be relevant today, may not have been identified in this review. Secondly, two databases were used with the aim of achieving more comprehensive coverage of published studies. Both searches generated results in English by chance. Articles published in other languages, as well as unpublished studies, may have been missed. Thirdly, most of the papers included were expert statements or opinions due to the nature of the topic. These were included to provide a broader range of perspectives on this controversial topic.

Another limitation is the geographical regions covered in this review: both Western and Eastern studies were included, though Western studies constituted the large majority (86%). It is important to evaluate ethical considerations from both Western and Eastern perspectives due to differing cultural backgrounds and the likely variation in clinical practices, ethical concerns, and approaches [17,18].

It should also be noted that the proportion of discussions dedicated to each ethical topic is not necessarily reflective of the size or importance of the dilemmas in practice.

With regard to the findings, most of the authors approached the ethical dilemmas from the framework of the four pillars of ethics, namely beneficence, non-maleficence, autonomy, and justice, alongside other ethical principles. Unsurprisingly, no definitive right or wrong answers could be concluded from the studies, as most authors presented opposing views due to the subjective and debatable nature of the topic. As conjoined twins involve two patients with intertwined interests, they should be analysed simultaneously. In many cases, the beneficence of one twin is therefore likely to signify maleficence to the other twin. It is also extremely challenging to achieve autonomy and justice for both twins at the same time. However, interesting viewpoints were mentioned in the papers, including the argument relating separation surgery to amputation and transplantation surgeries, as well as reasoning based on the concept of quality of life.

In amputations and transplantations, fewer dilemmas are noted, as the issues surrounding beneficence, non-maleficence, and consent appear to be less controversial. Some authors argued that separation surgery is comparable to involuntary amputation or transplantation, which we are more likely to disapprove of and therefore find less controversial. The key difference lies in the presence of another closely tied individual, which introduces additional factors to consider.

As third parties, we may be prone to analyse the best interests of patients based on external factors that are easily visible to us. However, the internalist theory offers an alternative perspective [19]. For individuals born as separate entities, the concept of being conjoined might seem equivalent to inconvenience and a loss of freedom. However, twins who have remained conjoined may hold different opinions, recognising possible benefits such as the close bond and intimacy shared with their twin.

More studies focusing on the ethics and possible approaches are needed to gain greater insight into the different ethical dilemmas healthcare professionals might face when considering separation surgery for conjoined twins. With more opinions and arguments made known, we can further discuss and learn from others’ strategies for addressing these concerns. This would not only build on current research evidence but also better prepare us for similar situations in future clinical practice.

## Conclusions

This paper provides an overview of the ethical controversies surrounding the separation surgery of conjoined twins. There are unlikely to be definitive right or wrong solutions to managing the ethical dilemmas raised. The importance lies in the process, whether we can address the various personal concerns or needs on a case-by-case basis, with guidance from the four pillars of ethics (beneficence, non-maleficence, justice, and autonomy) as well as the other principles discussed in this review. The relevant parties should be involved throughout the decision-making process, with good communication skills being a key element.
